# Association of serum steroids with survival in metastatic hormone-sensitive prostate cancer

**DOI:** 10.1530/ERC-24-0140

**Published:** 2025-01-10

**Authors:** Elahe A Mostaghel, Victoria Wang, Brett T Marck, Nima Sharifi, Alvin M Matsumoto, Christopher J Sweeney

**Affiliations:** ^1^ Geriatric Research, Education and Clinical Center, VA Puget Sound Health Care System, Seattle, Washington, USA; ^2^ Department of Medicine, Division of Oncology, University of Washington, Seattle, Washington, USA; ^3^ Fred Hutchinson Cancer Research Center, Washington, USA; ^4^ Dana-Farber Cancer Institute, Harvard Medical School, Boston, Massachusetts, USA; ^5^ Desai Sethi Urology Institute and Sylvester Comprehensive Cancer Center, University of Miami Miller School of Medicine, Miami, Florida, USA; ^6^ Department of Medicine, Division of Gerontology & Geriatric Medicine, University of Washington, Seattle, Washington, USA; ^7^ South Australian Immunogenomics Cancer Institute, University of Adelaide, Adelaide, Australia

**Keywords:** prostate cancer, hormone sensitive, chemohormonal therapy, steroid levels, docetaxel, E3805

## Abstract

The CHAARTED study showed that adding docetaxel (Doc) to androgen deprivation therapy (ADT) in men initiating treatment for metastatic hormone-sensitive prostate cancer (mHSPC) prolongs survival, particularly in high-volume disease. Androgens drive both mHSPC and metastatic castration-resistant prostate cancer (mCRPC). Lower nadir serum testosterone concentrations are associated with better outcomes in men treated with ADT for biochemical relapse, while higher androgens at mCRPC are associated with better prognosis and increased benefit from abiraterone. We evaluated the association of serum steroids at 24 weeks with overall survival (OS) and time to CRPC (TTCRPC) in 588 men with available samples from the CHAARTED study. Steroid concentrations were measured using mass spectrometry. The median testosterone concentration at 24 weeks was 8 ng/dL and did not differ in ADT alone vs ADT plus Doc arm. Achieving nadir testosterone below 20 ng/dL was not associated with OS or TTCRPC in either arm. In high-volume disease, Doc conferred an OS and TTCRPC benefit regardless of steroid concentrations. In low-volume disease, steroid concentrations in the lowest quartile at 24 weeks identified a subset of men with poor survival outcomes more like high-volume disease, and in whom Doc was also associated with improved OS and TTCRPC. The known OS benefit of Doc in high-volume mHSPC is not modified by serum steroid concentrations achieved on treatment. In low-volume disease, steroid concentrations in the lowest quartile may identify a poor prognosis subset in whom Doc also confers OS benefit.

## Introduction

Prostate cancer is the second most common cancer and the fifth leading cause of cancer death in men worldwide, with an estimated 1.4 million diagnoses and 375,000 deaths in 2020 (James *et al.* 2024). The incidence of prostate cancer varies by rates of prostate-specific antigen (PSA) screening and national guidelines, but cases of prostate cancer worldwide are projected to approach 3 million per year by 2040 (James *et al.* 2024). While localized and locally advanced prostate cancer is amenable to definitive therapy encompassing surgical, radiotherapy and focal ablative treatment modalities (Feng *et al.* 2023), recurrent or *de novo* metastatic prostate cancer requires systemic treatment approaches. Androgens are known drivers of prostate cancer, and inhibition of the androgen receptor (AR) signaling axis remains a mainstay of systemic therapy in metastatic hormone-sensitive prostate cancer (mHSPC) and metastatic castration-resistant prostate cancer (mCRPC). Next-generation AR signaling inhibitors in clinical practice include abiraterone, which inhibits adrenal steroid synthesis, and the AR inhibitors enzalutamide, apalutamide and darolutamide, which bind the AR and prevent nuclear translocation and transcriptional activation.

Lower nadir serum testosterone has been variably associated with better outcomes in men with biochemical relapse, locally advanced cancer or metastatic disease treated with androgen deprivation therapy (ADT), while higher androgens at the time of progression to CRPC have been associated with better prognosis and with better response to potent agents targeting the AR pathway (Claps *et al.* 2018). The CHAARTED study showed that adding docetaxel (Doc) to ADT in men initiating treatment for mHSPC prolongs survival, particularly in patients with high-volume disease (Kyriakopoulos *et al.* 2018), and this was confirmed by a meta-analysis (Vale *et al.* 2023). Whether chemotherapy influences the association of steroid levels with response to AR axis therapy is not well understood, nor whether steroid levels influence the association of tumor volume with response to chemotherapy.

We sought to determine whether response to hormonal vs chemohormonal therapy in patients with untreated metastatic prostate cancer is associated with serum androgen concentrations achieved on therapy, and whether this association is influenced by disease volume. We hypothesized that failure to achieve a nadir testosterone level below 20 ng/dL at 24 weeks after randomization in men commencing ADT for mHSPC conferred a shorter time to CRPC (TTCRPC) and a shorter overall survival (OS). Secondary analyses were performed to evaluate the association of steroid concentrations at 24 weeks and at the time of progression to CRPC with these outcomes in the ADT vs ADT plus docetaxel cohorts, and whether these associations differed in patient with high- vs low-volume disease.

## Materials and methods

### Study design and patient population

The CHAARTED trial (NCT00309985) enrolled patients with metastatic prostate cancer and randomized them 1:1 to either ADT alone or ADT with six cycles of docetaxel (given every 3 weeks). A total of 790 patients were enrolled between 2006 and 2012 across centers in the United States. The study was conducted in accordance with the Declaration of Helsinki for human subject protection. Samples were collected from patients who provided consent for the collection and storage of blood for future studies. The study group used for this analysis comprised trial patients who had serum samples available at study enrollment (referred to as baseline), at 24 weeks or at progression to CRPC. The Puget Sound Veterans Administration Institutional Review Board reviewed and approved the work conducted as part of this manuscript (#1588053).

### Procedures

Baseline clinicodemographic data at ADT initiation were obtained, such as age, ECOG performance status (PS), PSA and disease volume. High-volume disease was defined as the presence of visceral metastases and/or ≥ four bone metastases with at least one outside of the vertebral column and pelvis as based on CHAARTED criteria (Kyriakopoulos *et al.* 2018). Patients were allowed to have started ADT within 120 days of randomization. As testosterone levels begin to decline at 7–14 days of GnRH injection, but typically do not reach castrate levels until 21–28 days, baseline study samples were subdivided into those with less than vs greater than 2 weeks of ADT. While not a true baseline, samples collected at less than 2 weeks of ADT serve as a closer proxy of untreated serum levels than the baseline samples collected after 2 weeks. Outcome data were captured, including follow-up time, time to CRPC and OS. Serum steroid concentrations were measured by liquid chromatography–tandem mass spectrometry, as previously reported (Mostaghel *et al.* 2017). For purposes of calculation, serum steroid analyte values below the lower limit of detection were set at the lower limit of quantitation specific for that analyte.

### Study assessments

OS was defined as the time from randomization to death from any cause. TTCRPC was defined as the time from randomization until progression by PSA, imaging or symptoms. Survival from CRPC was defined as the time from progression (by imaging or symptoms) to death from any cause. Fourteen patients had not progressed to CRPC at the time clinical data were put together, and data at CRPC for these patients were set to not available.

### Statistical analysis

Comparisons of continuous variables among groups and between time points were assessed using the nonparametric Wilcoxon rank-sum test (Mann–Whitney test). Fisher’s exact test compared categorical variables. The Kaplan–Meier method estimated OS and time to CRPC. Hazard ratios (HRs) and confidence intervals were estimated using Cox proportional hazards regression models, and *P* values were two-sided, calculated using unstratified log-rank test without adjustment for multiplicity. Log-rank tests compared time to event distributions (e.g., OS and time to CRPC) between two groups. A testosterone level of 20 ng/dL (0.7 nmol/L) was used as a prespecified cutoff as this is the most common definition of castrate testosterone levels (Nishiyama 2014). Given the known discordance between testosterone measurement methods in castrated prostate cancer patients (Rouleau *et al.* 2019), we also categorized serum steroid levels by quartile, a commonly used cutoff that is likely to be more generally applicable in other datasets than any specific cutoff suggested by our study. The effects of steroids comparing the lowest quartile vs the three highest quartiles, and as a binary or continuous variable on OS, time to CRPC and survival from CRPC were assessed in multivariable Cox proportional models that adjusted for disease volume (high vs low).

### Study approval

Patients from the phase III CHAARTED study (NCT00309985) formed the study cohort and provided consent to the phase III study for treatment and gave permission for sample acquisition.

## Results

### Patient characteristics

Clinical data and samples from 588 patients from CHAARTED for whom serum was available at baseline, at 24 weeks or at the end of the study were included in the analysis. Patient clinicodemographic data were balanced between patients included in this study vs the overall cohort, except for PS for which the patients in this subset had a higher percentage of 0 (Table 1). A consort diagram is shown in Supplementary Data 1 (see the section on supplementary materials given at the end of the article).

**Table 1:** Clinical characteristics of patients included in this sub-study vs overall cohort.

Variable	Category	In	Out	Total	*P*-value
Total		588	202	790	–
Age	Mean (SD)	62.9 (8.7)	63.1 (8.9)	63.0 (8.7)	0.864
	Median (Q1, Q3)	63.0 (57.0,69.0)	63.0 (56.2,69.0)	63.0 (57.0,69.0)	
	[Min, Max]	[36.0,91.0]	[45.0,87.0]	[36.0,91.0]	
	Freq. of missing	0	0	0	
Race	White	510 (86.7)	164 (81.2)	674 (85.3)	0.069
	Black	51 (8.7)	25 (12.4)	76 (9.6)	
	Asian	9 (1.5)	1 (0.5)	10 (1.3)	
	Unknown	18 (3.1)	12 (5.9)	30 (3.8)	
	Unknown/missing	0	0	0	
ECOG PS	0	422 (71.9)	127 (62.9)	549 (69.6)	0.044
	1	157 (26.7)	72 (35.6)	229 (29.0)	
	2	8 (1.4)	3 (1.5)	11 (1.4)	
	Unknown/missing	1	0	1	
Volume	High	374 (63.6)	139 (68.8)	513 (64.9)	0.200
	Low	214 (36.4)	63 (31.2)	277 (35.1)	
	Unknown/missing	0	0	0	
Visceral disease	No	281 (75.3)	108 (77.7)	389 (76.0)	0.642
	Yes	92 (24.7)	31 (22.3)	123 (24.0)	
	Unknown/missing	215	63	278	
Gleason	<8	170 (32.1)	51 (29.0)	221 (31.3)	0.454
	8+	359 (67.9)	125 (71.0)	484 (68.7)	
	Unknown/missing	59	26	85	
Baseline PSA	Mean (SD)	367.5 (884.7)	395.9 (984.4)	374.8 (910.6)	0.074
	Median (Q1, Q3)	49.5 (12.4,261.2)	64.5 (18.4,269.6)	51.4 (13.7,266.1)	
	[Min, Max]	[0.1,8056.0]	[0.3,8540.1]	[0.1,8540.1]	
	Freq. of missing	4	2	6	
Prior adjuvant HT	No	563 (95.9)	192 (95.0)	755 (95.7)	0.688
	Yes	24 (4.1)	10 (5.0)	34 (4.3)	
	Unknown/missing	1	0	1	
Prior local treatment	No local therapy	415 (70.7)	160 (79.2)	575 (72.9)	0.057
	Prostatectomy	125 (21.3)	29 (14.4)	154 (19.5)	
	Primary radiation	47 (8.0)	13 (6.4)	60 (7.6)	
	Unknown/missing	1	0	1	

PS, performance status; PSA, prostate-specific antigen.

### Serum steroid levels in the ADT and ADT plus docetaxel cohorts

**Figure 1:**
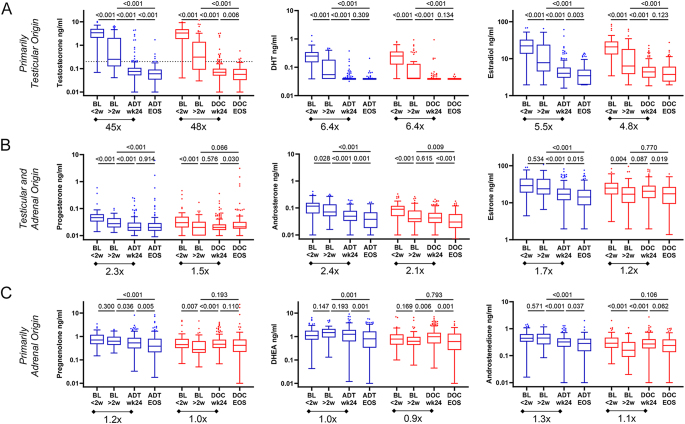
Within-group comparison of steroid levels at each time point in ADT alone (blue) vs ADT + docetaxel (red) treatment arms. Steroids are grouped according to whether they are primarily (A) of testicular origin (testosterone, DHT and estradiol), (B) of mixed testicular and adrenal origin (progesterone, androsterone and estrone) or (C) of primarily adrenal origin (pregnenolone, DHEA and AED). Data are shown as box-and-whisker plots, where the horizontal lines indicate median values; the white boxes denote the 75th (upper margin) and 25th percentiles (lower margin), and the upper and lower bars indicate the minimum and maximum values, respectively. BL, baseline; wk, week; ADT, androgen deprivation therapy; Doc, docetaxel; and EOS, end of study.

Steroid levels were measured in samples taken at baseline, at 24 weeks of treatment and at the end of the study (at the time of progression to CRPC). In baseline samples taken within 2 weeks of starting ADT (at which point serum testosterone is only partially suppressed from baseline), older age was associated with lower levels of the primary adrenal androgen (DHEA) but not the primary testicular androgens (testosterone) (Supplementary Data 2A). This is consistent with the known decline in adrenal steroid production associated with aging, while data show a lack of age-related declines in testicular steroids (Cappola *et al.* 2023). In contrast, after 24 weeks of treatment, the lower levels of steroids in both categories were associated with older age, consistent with the adrenal gland becoming the only source of androgen production in patients on ADT (Supplementary Data 2B). Steroid levels did not associate with ECOG PS (1 vs 0; not shown). The median steroid levels at each time point for patients in the ADT vs ADT plus docetaxel arms are summarized in Supplementary Data 3 and are shown in Fig. 1. The relationship of the measured sex steroids to each other in the steroidogenic pathway is shown in Supplementary Data 4. The steroids in Fig. 1 are grouped according to whether they are primarily of testicular origin (Fig. 1A; testosterone, DHT and estradiol), of mixed testicular and adrenal origin (Fig. 1B; progesterone, androsterone and estrone) or of primarily adrenal origin (Fig. 1C; pregnenolone, DHEA and AED).

As expected, the levels of the primarily testicular steroids testosterone, DHT and estradiol were significantly inhibited by ADT. Substantial and similar decreases from baseline (samples collected at less than two weeks of ADT therapy) to the 24-week time point were observed in both the ADT alone and ADT plus docetaxel treatment arms (∼45-fold, ∼6-fold and ∼5-fold decreases for testosterone, DHT and estradiol, respectively (Fig. 1A)), with a median testosterone level of 0.08 ng/mL (8 ng/dL) or less by week 24. These three steroids also demonstrated significant additional declines in the baseline samples collected at greater than vs less than two weeks of ADT therapy. In contrast, the steroids of mixed testicular and adrenal origin, including progesterone, androsterone and estrone, show a more modest decrease (Fig. 1B; ∼1.5- to 2.3-fold from baseline to week 24), while the levels of the primarily adrenal steroids pregnenolone, DHEA and AED are minimally affected (Fig. 1C; ∼0.9- to 1.3-fold decrease from baseline to week 24). Steroid levels were not increased at progression to CRPC in either treatment group.

Comparison of the ADT alone vs ADT plus docetaxel treatment groups shows no difference in the levels of the primarily testicular steroids testosterone, DHT or estradiol at any time point (Supplementary Data 5A). Notably, however, the levels of the mixed origin and primarily adrenal steroids are consistently lower in baseline samples from the ADT plus docetaxel vs ADT only treatment group (Supplementary Data 5B, 5C). This difference persists at week 24 for androsterone, estrone, DHEA and AED, but by the final time point, there is no longer any difference in the two treatment groups. The enhanced suppression of these steroid levels in the docetaxel treatment arm is consistent with the fact that most baseline samples were collected on the day chemotherapy was started (Supplementary Data 5D) and, therefore, after the receipt of pretreatment dexamethasone, which would have led to the suppression of adrenal steroid synthesis.

### Correlations of steroid levels in the ADT and ADT plus docetaxel cohorts

**Figure 2:**
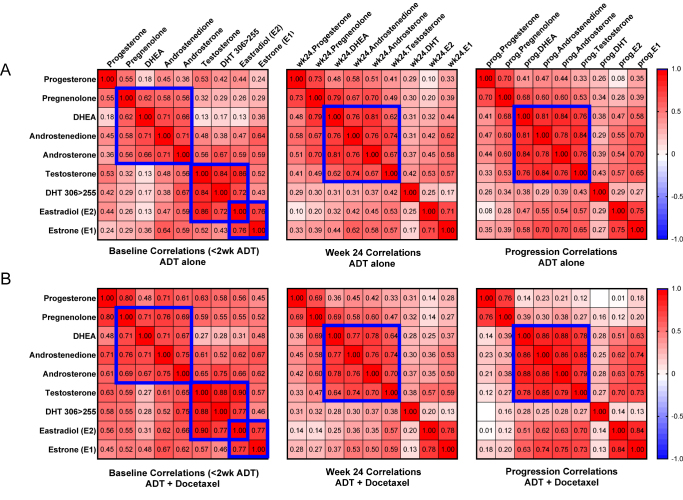
Correlation of steroid levels at each time point in the ADT alone and the ADT plus docetaxel treatment arms. Spearmen correlation of steroid levels in (A) the ADT alone and (B) the ADT plus docetaxel treatment arms at the indicated time point. Correlations are color-coded from highly directly correlated (dark red) to highly indirectly correlated (dark blue). The blue boxes indicate the relationships within adrenally derived steroids and within testicular-derived steroids and the increased correlation of testosterone with the adrenal steroids at each time point. ADT, androgen deprivation therapy.

Steroid correlations at each time point were very similar in the ADT alone and ADT plus docetaxel arms (Fig. 2A and B). At baseline (samples collected at less than two weeks of ADT therapy), Spearman correlation coefficients were very high among the adrenal steroids pregnenolone, DHEA, androstenedione and androsterone; among the testicular steroids testosterone, DHT and estradiol; and between estradiol and estrone (Fig. 2, blue squares, left panels). Estradiol and estrone were highly correlated with each other at all time points. Estradiol was otherwise consistently highly correlated with its direct precursor testosterone, while estrone was more highly correlated with its precursor androstenedione. Testosterone became progressively more correlated with the adrenal steroids at each time point (Fig. 2, blue squares, middle and right panels), consistent with the fact that in the castrate setting, circulating testosterone becomes derived from adrenal precursors.

### Association of HSD3B1 genotype with steroid levels

The adrenal permissive 1245C allelic variant of *HSD3B1* produces a more stable enzyme, increasing the conversion rate of DHEA to AED, a precursor to the production of testosterone and DHT (Supplementary Data 4). The 1245C variant has been associated with earlier development of castration-resistance and shorter OS in men with low-volume metastatic prostate cancer treated on the CHAARTED study (Chang *et al.* 2013, Hearn *et al.* 2020), but association with serum androgen levels has not been observed (Serag *et al.* 2021). We likewise found no consistent decrease in serum levels of steroids immediately upstream of this enzyme (pregnenolone and DHEA) or increase in steroids immediately downstream of this enzyme (progesterone, androstenedione and testosterone) in carriers of 1 or 2 variant *HSD3B1* alleles at baseline or during treatment (Supplementary Data 6).

### Association of treatment arm with outcome in patients with low and high steroid levels

Achievement of a nadir testosterone level below the specified cutoff of 20 ng/dL (0.7 nmol/L) at week 24 showed no association with OS or TTCRPC in either the ADT alone arm or the ADT plus docetaxel treatment arm (Supplementary Data 7), likely because the median testosterone level at this time point was 0.08 ng/mL (8 ng/dL), well below the 20 ng/dL cutoff. Therefore, we categorized serum steroid levels by quartile, comparing the lowest quartile (Q1) to the highest three quartiles (Q234). For testosterone, this was patients with values ≤0.051 ng/mL vs >0.051 ng/mL at 24 weeks and values ≤0.034 ng/L vs >0.034 ng/mL at progression. Marker distributions for all steroid levels at week 24 and progression are shown in Supplementary Data 8.

**Figure 3:**
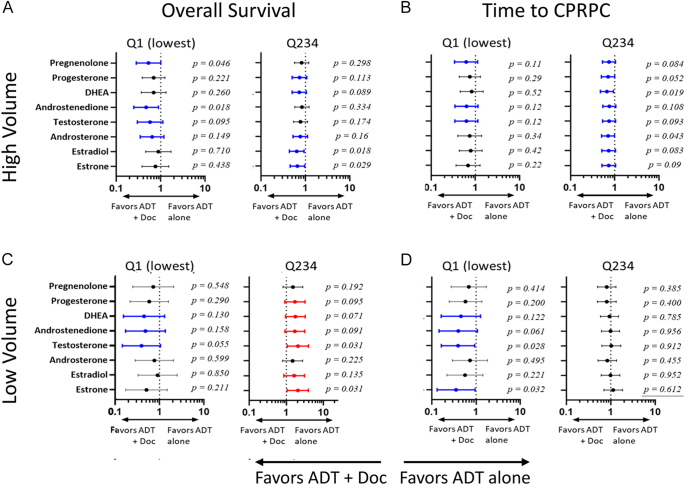
Association of treatment arm with the outcome in patients with steroid levels in the lowest (Q1) or three highest (Q234) quartiles stratified by disease volume. Forest plot of unadjusted HR associated with treatment with ADT + Doc vs ADT alone for (A) OS in high-volume disease, (B) time to CRPC in high-volume disease, (C) OS in low-volume disease and (D) time to CRPC in low-volume disease. HR colored for *P* ≤ 0.15: HR in blue favors ADT + Doc; HR in red favors ADT alone. Q1, lowest quartile; Q234, highest three quartiles; HR, hazard ratio; ADT, androgen deprivation therapy; Doc, docetaxel; OS, overall survival; and CRPC, castration-resistant prostate cancer.

We first evaluated the impact of treatment with ADT alone vs ADT plus docetaxel in patients in the lowest quartile (Q1) vs the highest 3 quartiles (Q234) of steroid levels at week 24. The unadjusted HRs for OS and TTCRPC by disease volume and level (Q1 vs Q234) of each steroid are shown in Fig. 3 and summarized in Supplementary Data 9.

As expected, OS and TTCRPC in patients with high-volume disease were favored in patients treated with ADT plus docetaxel (Fig. 3A and B), and the benefit of docetaxel was observed in terms of whether patients were in the lowest (Q1) or highest three (Q234) quartiles of steroid levels. Unexpectedly, in low-volume patients, steroid levels in Q1 appear to identify a subset in whom OS and TTCRPC were also favored by ADT plus docetaxel (Fig. 3C and D, left side panels). In the remaining majority of patients with low-volume disease (the highest three quartiles of steroid levels), OS and TTCRPC were not favored by treatment with docetaxel. Notably, in these low-volume patients with steroid levels in the highest three quartiles, OS was actually favored by treatment with ADT alone (Fig. 3C, right panel). Survival from CRPC was not consistently favored in either treatment arm (Supplementary Data 9).

These findings demonstrate no impact of steroid levels on the known OS benefit associated with docetaxel in patients with high-volume disease. However, the observations suggest that in patients with low-volume disease, having steroid levels in the lowest quartile may identify an additional subset of patients who would also derive clinical benefit from docetaxel.

### Association of steroid levels with outcome in patients treated with ADT alone or ADT plus docetaxel

**Figure 4:**
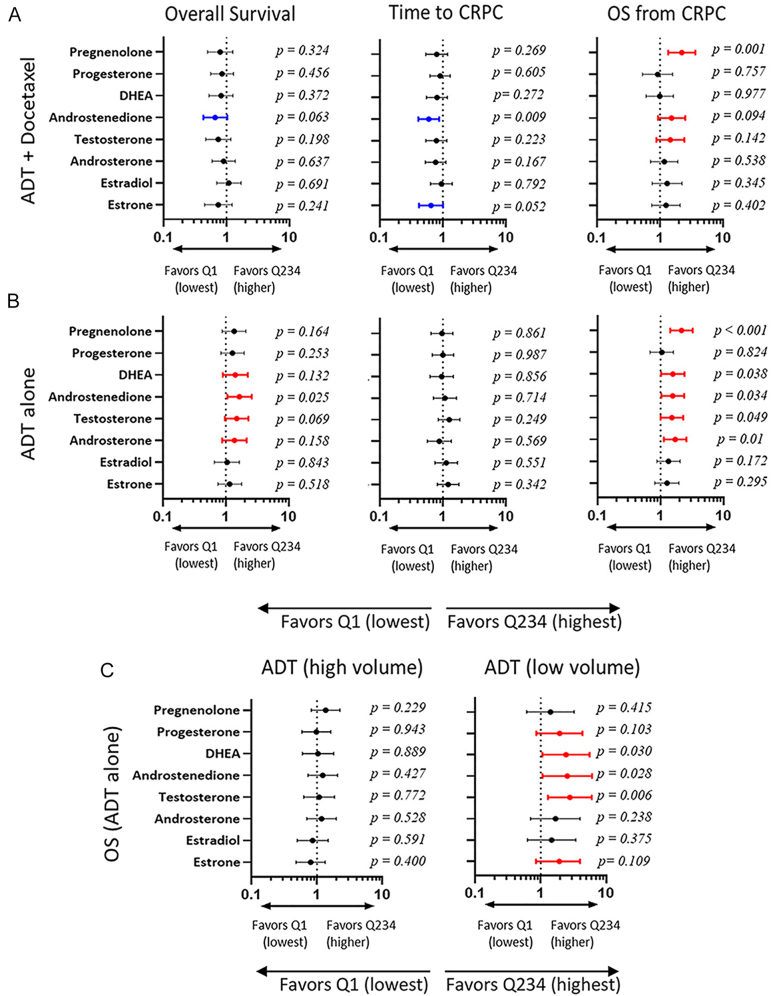
Association of steroid levels with outcome in patients treated with ADT alone or ADT plus docetaxel. Forest plots of unadjusted HR for OS, time to CRPC and OS from CRPC associated with having levels of the indicated steroids in Q1 vs Q234 in patients treated with (A) ADT + docetaxel or (B) ADT alone. (C) Forest plots of unadjusted HR for OS associated with having levels of the indicated steroid in Q1 vs Q234 in ADT-treated patients with high- or low-volume disease. HR colored for *P* ≤ 0.15: HR in blue favors Q1; HR in red favors Q234. ADT, androgen deprivation therapy; HR, hazard ratio; OS, overall survival; CRPC, castration-resistant prostate cancer; Q1, lowest quartile; and Q234, highest three quartiles.

In prior studies, lower nadir testosterone levels on ADT have been associated with better outcomes, while higher androgens at progression to mCRPC have been associated with better prognosis and better response to AR-targeted therapy. We therefore evaluated the impact of steroid levels in the lowest quartile (Q1) vs the highest three quartiles (Q234) on OS, TTCRPC, and OS from CRPC, separately in each treatment arm. The unadjusted HRs associated with having steroid levels in Q1 vs Q234 in the ADT plus docetaxel and ADT alone arms are shown in Fig. 4 and summarized in Supplementary Data 10.

A consistent association of steroid levels with OS or TTCRPC was not observed in the ADT plus docetaxel arm (Fig. 4A), nor with TTCRPC in the ADT alone arm (Fig. 4B). In the ADT alone arm (Fig. 4B), higher steroid levels at week 24 of ADT alone were consistently associated with improved OS. Higher steroid levels at progression to CRPC were also consistently associated with better OS from CRPC in the ADT alone arm (consistent with prior observations) and, to a lesser extent, in the ADT plus docetaxel arm.

**Figure 5:**
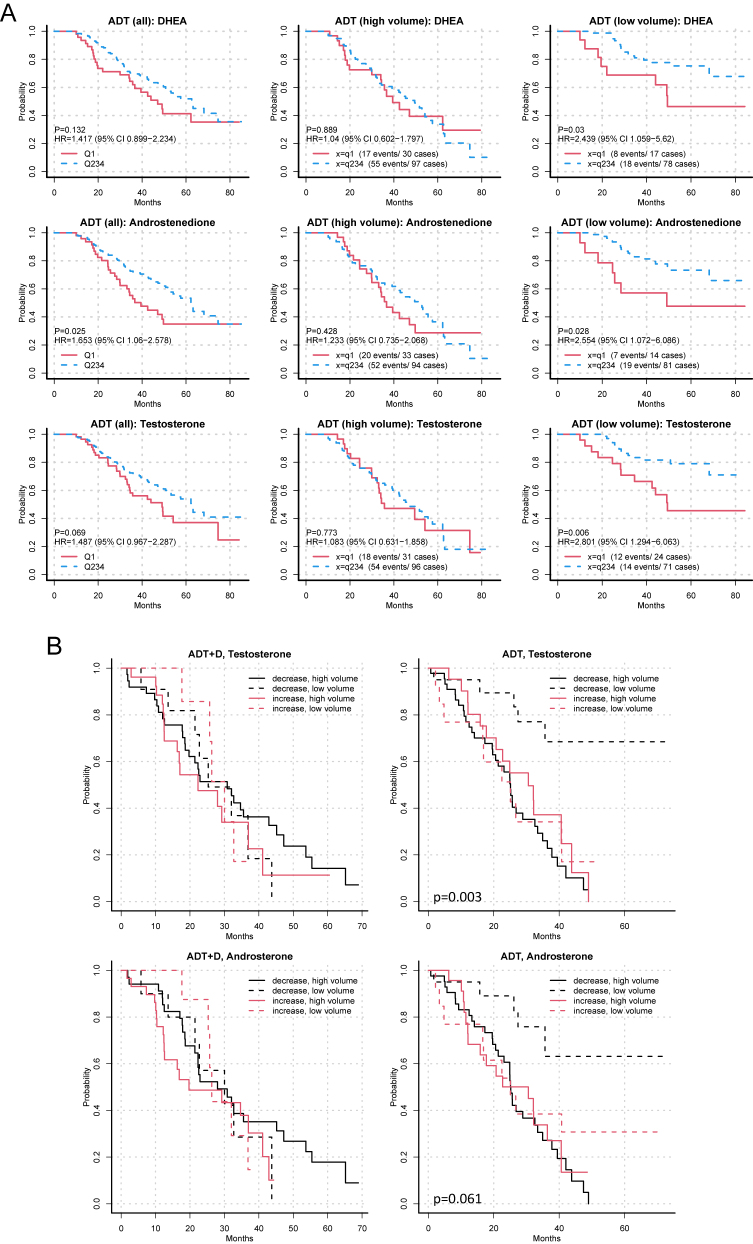
Overall survival curves associated with steroid levels stratified by high- and low-volume disease. (A) OS curves associated with having DHEA, AED and testosterone levels in the lowest (Q1) vs three highest (Q234) quartiles in all patients treated with ADT alone, or in patients separated by high- vs low-volume disease. (B) OS from CRPC associated with change in testosterone or androsterone from week 24 to CRPC (increase – red; decrease – black) in patients treated with ADT plus docetaxel or ADT alone stratified by disease volume (low – dashed; high – solid). OS, overall survival; Q1, lowest quartile; Q234, highest three quartiles; ADT, androgen deprivation therapy; and CRPC, castration resistant prostate cancer.

Further evaluation of the HRs for OS in the ADT alone arm shows that the OS benefit associated with higher steroid levels at week 24 is limited to patients with low-volume disease (Fig. 4C). The OS curves associated with the levels of DHEA, AED and testosterone in the lowest (Q1) vs highest three (Q234) quartiles are shown in Fig. 5A, also demonstrating that the benefit associated with higher steroid levels (Q234) at week 24 is present only in patients with low-volume disease. Notably, in low-volume patients, the survival curves associated with having steroid levels in the lowest quartile (Q1) are more like the survival curves in high-volume patients, consistent with the observations in Fig. 3D, suggesting that low levels at week 24 are associated with a poor prognosis and possibly predictive of a clinical benefit for docetaxel in this patient subset.

Finally, we evaluated whether OS was associated with increasing or decreasing steroid levels at the time of progression to CRPC. In the ADT plus docetaxel arm, there were no differences in survival associated with increasing or decreasing levels of testosterone, AED, DHEA or androsterone from week 24 to CRPC in the ADT plus docetaxel arm (Fig. 5B and not shown). In the ADT alone arm, there were no associations with AED or DHEA; however, survival was substantially prolonged in those with low-volume disease and decreasing levels of testosterone or androsterone from week 24 to CRPC (Fig. 5B; broken black lines). In contrast, those with low-volume disease and increasing levels of testosterone or androsterone (broken red lines) had survival curves indistinguishable from high-volume disease patients, consistent with the worse OS observed in Fig. 5A for low-volume disease patients who were in the lowest quartile of testosterone and androsterone levels at week 24.

## Discussion

The CHAARTED study showed that adding docetaxel to ADT in men initiating treatment for mHSPC prolongs survival, particularly in patients with high-volume disease (Kyriakopoulos *et al.* 2018). Our study demonstrates that the OS benefit of docetaxel in high-volume disease is observed regardless of whether patients achieve on-treatment steroid levels in the lowest (Q1) or highest three (Q234) quartiles. However, in those with low-volume disease, those with steroid levels in the lowest quartile (Q1) at week 24 appear to comprise a subset in whom OS and TTCRPC are also favored by the addition of docetaxel. These findings suggest that being in the lowest quartile of steroids levels is both a poor prognostic marker while on treatment and a post-treatment predictor of benefit from docetaxel in mHSPC patients with low-volume disease.

Both prospective and retrospective data have suggested a relationship between achieving testosterone levels below the historical standards of 1.7 nmol/L (50 ng/dL) or 0.7 nmol/L (20 ng/dL) and an improvement in outcomes, suggesting that reaching a threshold level of androgen suppression is important in optimizing the response to ADT (Attard *et al.* 2009, Ryan *et al.* 2013, Kim *et al.* 2014, Klotz *et al.* 2017, Claps *et al.* 2018, Suzuki *et al.* 2018, Miura *et al.* 2020). In the present study, achievement of a nadir testosterone level below the specified cutoff of 20 ng/dL (0.7 nmol/L) at week 24 showed no association with OS or TTCRPC in either the ADT alone arm or the ADT plus docetaxel treatment arm, likely because the median testosterone level at this time point was 0.08 ng/mL (8 ng/dL), well below the 20 ng/dL cutoff. In this regard, a majority of prior studies used ELISA, well documented to be inaccurate and to overestimate testosterone levels in the sub-nanomolar range due to cross-reactivity with other steroids (Moal *et al.* 2007).

Importantly, once below this threshold of testosterone suppression, the relative level of residual androgens available for uptake in the tumor microenvironment may confer a prognostic difference in disease biology, wherein disease progression in the lowest steroid environments may facilitate the outgrowth of adverse tumor biology. This is consistent with the observation that lower serum androgen levels at the time of progression to CRPC were associated with decreased survival in both the placebo and abiraterone acetate arms of the phase III study of abiraterone acetate plus prednisone in men with metastatic CRPC (Ryan *et al.* 2013), and with a meta-analysis demonstrating that PFS and OS were lower in CRPC patients with lower vs higher testosterone levels (Miura *et al.* 2020).

While no association was observed in the ADT plus docetaxel arm, in the ADT alone arm steroid levels in the highest quartiles at week 24 and at progression to CRPC were consistently associated with improved OS, and OS from CRPC, respectively, with the OS benefit clearly limited to patients with low-volume disease. These findings suggest that having on-treatment steroids levels in the highest quartiles is prognostic for improved OS in mHSPC patients with low-volume disease. Conversely, the treatment curves for patients with steroid levels in the lowest quartile (Q1) more closely approximated the survival curves in high-volume disease patients, suggesting that having on-treatment steroids levels in the lowest quartile (Q1) is an adverse prognostic factor for mHSPC patients with low-volume disease.

The adrenal permissive 1245C *HSD3B1* allele codes for a protein variant with increased stability and subsequently increased synthesis of potent androgens from extragonadal precursors. It has been associated with worse outcomes after ADT (Thomas & Sharifi 2020), including in men with low-volume mHSPC in CHAARTED (Hearn *et al.* 2020). In contrast, upfront treatment with direct AR blockade in addition to ADT in the ENZAMET trial reversed the adverse association of the 1245C *HSD3B1* variant and was associated with improved OS in men with low-volume mHSPC, consistent with an enhanced effect of this variant on tumor steroids and AR signaling in low-volume cancers (Sharifi *et al.* 2024).

Consistent with prior report, we did not observe a specific association of this allelic variant with serum androgen levels, suggesting that the salient effect of this variant is at the tissue level (Serag *et al.* 2021). Together, these studies are consistent with a model in which *HSD3B1* expression and activity are largely present in peripheral tissues (including prostate cancer) without any major association between genotypes and circulating androgens.

Our findings are consistent with a hypothesis in which low ambient steroids adversely influence the behavior of low-volume disease in patients treated with ADT. Of note, while lower androgen levels were associated with more advanced age in this analysis, the age-related decline in adrenal androgen levels shows high inter-individual variability (Parker 1999). Thus, while the lowest steroid levels will tend to be present in older men, not all older men will have steroid levels in the lowest quartile. Low steroid levels may have minimal impact on the more aggressive biology that characterizes high volume disease. They may also have minimal impact in the context of therapies which do not primarily target AR signaling, such as docetaxel (Mostaghel 2013, Sutera *et al.* 2022). Instead, low steroid levels may be an indicator of which low volume patients treated with AR-directed therapy are likely to progress with poor biology. This may include upregulation of non-AR pathways, explaining why patients with otherwise favorable low-volume disease derive benefit from docetaxel. It may also include upregulation of AR, explaining the more universal benefit associated with the second-generation AR pathway inhibitors seen in mHSPC (Lolli *et al.* 2019).

However, the biological basis for the impact of low steroid levels on tumor biology is unclear, and given this is a post-treatment variable, it cannot be defined *a priori*. Moreover, the patients in the lowest quartile at week 24 may have been in the lowest quartile at baseline, but the collection of blood in most patients after starting ADT prohibits assessing this correlation. Other efforts to correlate the baseline biological variables (e.g., germline genetic profiles) and their association with low steroid levels may identify men with good prognosis by disease volume on scans who have a more aggressive underlying biology and who would benefit from treatment intensification with docetaxel. Whether this holds true in the face of the current standard of care for mHSPC being ADT plus abiraterone, enzalutamide or darolutamide with or without docetaxel is unknown.

Notably, most studies linking low testosterone levels on ADT to improved outcomes were prior to the approval of potent AR pathway inhibitors. However, in the context of next-generation AR pathway inhibitors, higher androgen levels at CRPC have been associated with improved responses to therapy. In the low-volume disease patients in CHAARTED, which had a cutoff date for survival of April 23, 2016, all patients would have had access to next-generation AR-targeted agents at progression, potentially contributing to the association of improved OS in low-volume ADT-treated patients with higher androgen levels.

This study has several important limitations. Pretreatment (eugonadal) steroid levels have also been previously associated with outcomes on ADT, but we were unable to address this question as baseline values in this study were mandated to be within three weeks of starting ADT, not prior to ADT. Biopsy studies sampling metastatic tissues before and after therapy have also associated low tumor steroid levels with worse outcomes in response to AR-directed therapy, but these have been in the mCRPC setting and data in the mHSPC setting are lacking (Mostaghel *et al.* 2021). The lack of tissue-based molecular profiles (e.g., as evaluated via single-cell technologies) that could shed light on the association of disease biology with steroid levels is clearly a limitation in interpreting the data in this study (Feng *et al.* 2024). Finally, no correction was made for multiple testing. Many of the steroid metabolites evaluated in this study are highly correlated such that the findings cannot be taken as independent observations.

While these data must be considered hypothesis generating, the assessment of multiple correlated metabolites does provide an opportunity to judge the robustness of the findings. For correlated metabolites such as the serum steroids in this study, a biologically meaningful association with one metabolite would be expected to be consistently evident across most of the related metabolites, whereas an outcome associated with only one or two metabolites is expected to be more likely related to chance. In this regard, the associations called out in this study were consistently and similarly observed for DHEA, androstenedione, testosterone and androsterone. Practically, this suggests that future studies might reasonably focus on the assessment of a more limited number of steroids, such as testosterone, and an adrenal androgen, such as DHEA, rather than an expanded panel. However, standardization of assay methods and clinical coordination to facilitate widespread access to state-of-the-art laboratory equipment remains necessary for optimal steroid measurements.

We demonstrate that the known OS benefit of docetaxel in high-volume mHSPC is not modified by steroid levels achieved on treatment. However, in patients with low-volume disease, having steroid levels in the lowest quartile at week 24 may identify a poor prognosis subset in whom docetaxel would provide an OS benefit.

## Supplementary materials



## Declaration of interest

EA Mostaghel reports research support from ESSA Pharma and Propella Therapeutics. N Sharifi reports pending patents related to *HSD3B1*. Christopher J Sweeney reports playing consulting or advisory role for Sanofi, Janssen, Astellas Pharma, Bayer, Genentech, AstraZeneca, Pfizer and Celgene. Research funding was given by Janssen Biotech (Inst), Astellas Pharma (Inst), Sanofi (Inst), Bayer (Inst), Sotio (Inst) and Dendreon (Inst); patents, royalties, and other intellectual properties were given by parthenolide (Indiana University); dimethylaminoparthenolide (Leuchemix); and abiraterone plus cabozantinib combination (Exelixis). Stock or other ownership was held by Leuchemix. The remaining authors declare that there is no conflict of interest that could be perceived as prejudicing the impartiality of the research reported.

## Funding

This study was supported by the National Cancer Institute of the National Institutes of Healthhttps://doi.org/10.13039/100000002 (NIH) under the following award numbers: U10CA180820, U10CA180794, UG1CA233180, UG1CA233196 and R01CA208254-01 and by Sanofi.
