# Supplementary material for: Association of serum steroids with survival in metastatic hormone-sensitive prostate cancer
Source: Endocr Relat Cancer. 2025 Jan 10;32(2):e240140. doi: 10.1530/ERC-24-0140 (PMC11798412; doi:10.1530/ERC-24-0140)
Supplement: Supplementary file 1 [file supplementary_materials.pdf]

Supplementary Data 1 – Consort Diagram

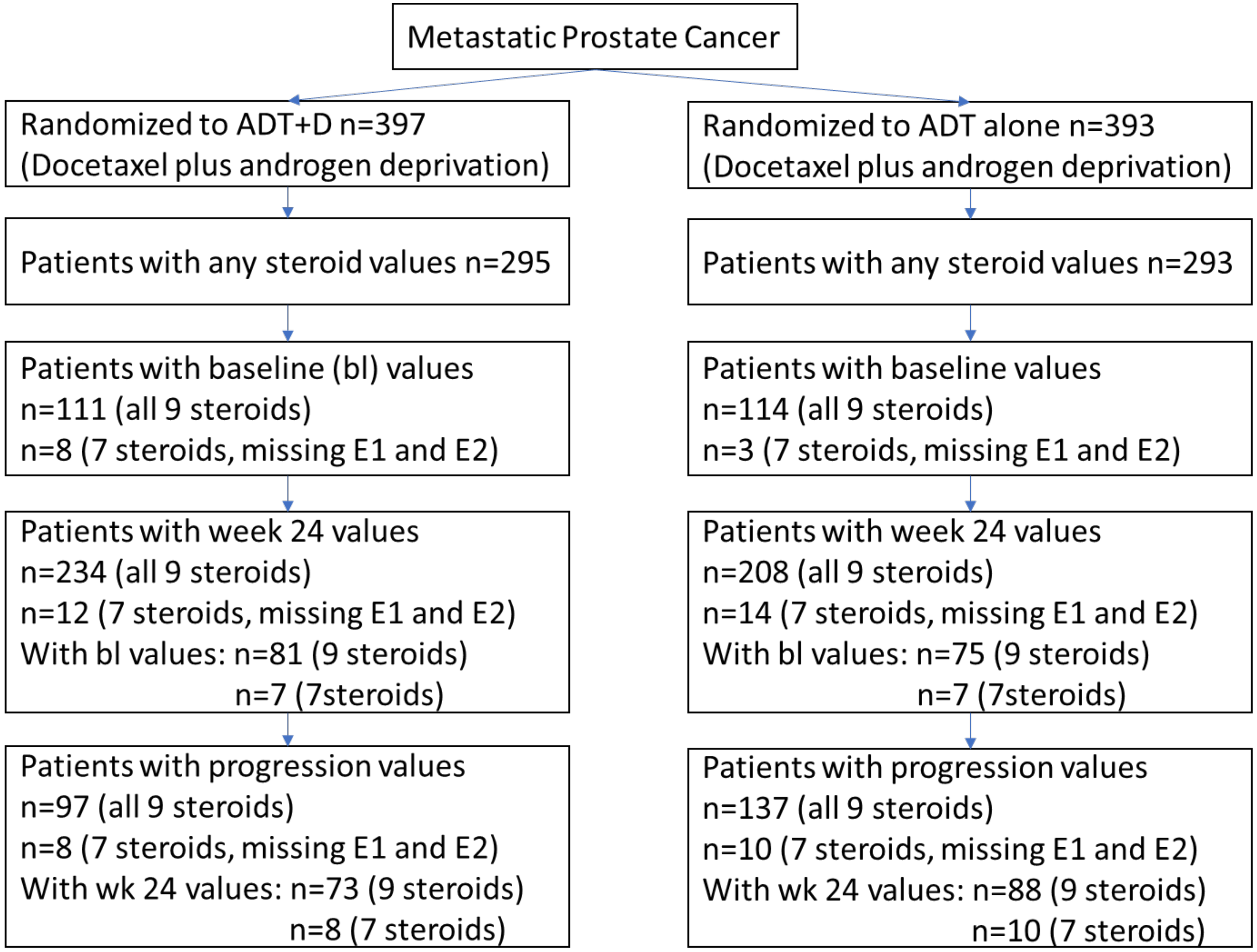

Supplementary Data 2 – Patient age by baseline (0-2 weeks of ADT) or week 24 hormone levels (Q1 vs Q234). P-value from Wilcoxon test.

By steroid levels at baseline (0-2 weeks of ADT)

Variable	Category	Q1, mean (SD)	Q234, mean (SD)	P-value
Androstenedione	All	63.5 (10.6)	63.2 (8.3)	0.840
	ADT+D	63.9 (11.0)	62.9 (6.7)	0.678
	ADT	62.0 (9.7)	63.3 (9.2)	0.766
Androsterone	All	67.2 (8.4)	61.9 (8.7)	0.005
	ADT+D	67.2 (8.9)	61.5 (8.0)	0.037
	ADT	67.2 (7.9)	62.1 (9.2)	0.066
DHEA	All	69.7 (7.3)	61.1 (8.3)	< 0.001
	ADT+D	69.2 (7.4)	60.5 (7.7)	< 0.001
	ADT	70.6 (7.5)	61.6 (8.7)	0.003
DHT	All	63.6 (10.0)	63.1 (8.5)	0.910
	ADT+D	63.7 (11.7)	63.1 (7.1)	0.988
	ADT	63.6 (7.6)	63.1 (9.6)	0.951
Pregnenolone	All	63.2 (10.4)	63.3 (8.4)	0.854
	ADT+D	63.4 (11.0)	63.2 (7.3)	0.956
	ADT	62.6 (9.6)	63.3 (9.2)	0.819
Progesterone	All	61.7 (10.4)	63.8 (8.3)	0.197
	ADT+D	62.2 (11.3)	63.9 (6.6)	0.380
	ADT	60.3 (8.2)	63.7 (9.3)	0.279
Testosterone	All	63.9 (10.4)	63.0 (8.3)	0.748
	ADT+D	63.4 (12.0)	63.2 (7.1)	0.837
	ADT	64.5 (8.6)	62.8 (9.4)	0.562
E2	All	61.8 (9.8)	63.6 (8.6)	0.131
	ADT+D	63.3 (12.4)	63.2 (7.3)	0.747
	ADT	60.2 (6.4)	64.0 (9.8)	0.072
E1	All	62.9 (6.4)	63.2 (9.7)	0.532
	ADT+D	64.1 (6.7)	62.9 (9.5)	0.850
	ADT	61.5 (5.8)	63.6 (10.0)	0.267

Distribution of age (all patients regardless of whether hormone data are available):
All: mean 62.9, SD 8.7
ADT+D: mean 63.6, SD 8.5
ADT: mean 62.3, SD 8.9

By steroid levels at week 24 of ADT

Variable	Category	Q1, mean (SD)	Q234, mean (SD)	P-value
Androstenedione	All	66.8 (8.4)	61.8 (8.0)	< 0.001
	ADT+D	67.9 (8.1)	61.9 (7.9)	< 0.001
	ADT	65.1 (8.7)	61.7 (8.1)	0.009
Androsterone	All	66.7 (8.5)	61.8 (7.9)	< 0.001
	ADT+D	66.9 (8.5)	62.3 (8.0)	< 0.001
	ADT	66.4 (8.7)	61.3 (7.9)	< 0.001
DHEA	All	68.4 (8.0)	61.3 (7.7)	< 0.001
	ADT+D	68.5 (7.8)	61.8 (7.8)	< 0.001
	ADT	68.3 (8.3)	60.8 (7.6)	< 0.001
DHT	All	63.5 (8.4)	59.1 (7.3)	< 0.001
	ADT+D	64.1 (8.3)	58.5 (7.7)	0.004
	ADT	62.8 (8.4)	59.6 (7.2)	0.042
Pregnenolone	All	66.7 (7.8)	61.9 (8.2)	< 0.001
	ADT+D	67.8 (6.6)	62.4 (8.5)	< 0.001
	ADT	65.6 (8.7)	61.3 (7.9)	< 0.001
Progesterone	All	64.2 (8.5)	62.6 (8.3)	0.094
	ADT+D	64.8 (7.5)	63.2 (8.7)	0.215
	ADT	63.5 (9.6)	62.0 (7.8)	0.309
Testosterone	All	65.6 (8.5)	62.2 (8.1)	< 0.001
	ADT+D	66.2 (8.2)	62.8 (8.2)	0.002
	ADT	65.0 (8.9)	61.6 (8.0)	0.013
E2	All	62.8 (9.4)	63.1 (8.0)	0.576
	ADT+D	62.4 (9.6)	63.8 (7.9)	0.296
	ADT	63.1 (9.2)	62.3 (8.1)	0.741
E1	All	63.6 (9.3)	62.9 (8.1)	0.302
	ADT+D	63.5 (9.7)	63.5 (8.0)	0.903
	ADT	63.7 (9.0)	62.1 (8.1)	0.142

Supplementary Data 3 – Distribution of indicated steroid levels over time in each treatment arm

Testosterone	ADT				ADT + Doc			
	BL<2w	BL>2w	wk24	EOS	BL<2w	BL>2w	wk24	EOS
Minimum	0.069	0.041	0.01	0.01	0.04	0.03	0.01	0.01
25% Percentile	2.07	0.125	0.0518	0.034	1.85	0.09	0.05	0.0325
Median	3.39	0.251	0.08	0.06	3.33	0.31	0.07	0.057
75% Percentile	4.99	2.06	0.108	0.091	4.8	1.4	0.0995	0.097
Maximum	10.3	6.7	4.46	1.7	9.78	11.8	3.26	0.571
Mean	3.58	1.47	0.167	0.0829	3.51	1.34	0.13	0.0751
Std. Deviation	2.07	1.96	0.502	0.146	2.26	2.37	0.356	0.0722

Dihydrotestosterone (DHT)	ADT				ADT + Doc			
	BL<2w	BL>2w	wk24	EOS	BL<2w	BL>2w	wk24	EOS
Minimum	0.039	0.039	0.032	0.039	0.04	0.04	0.039	0.039
25% Percentile	0.15	0.039	0.039	0.039	0.125	0.04	0.039	0.039
Median	0.249	0.055	0.04	0.039	0.25	0.04	0.039	0.039
75% Percentile	0.344	0.186	0.039	0.039	0.36	0.133	0.039	0.039
Maximum	1.31	0.471	0.377	0.207	0.81	0.93	0.935	0.057
Mean	0.276	0.129	0.0436	0.0412	0.26	0.134	0.0454	0.0394
Std. Deviation	0.2	0.13	0.0272	0.0146	0.167	0.18	0.0598	0.00211

Estradiol (E2)	ADT				ADT + Doc			
	BL<2w	BL>2w	wk24	EOS	BL<2w	BL>2w	wk24	EOS
Minimum	1.96	1.96	1.6	1.96	3.44	1.96	1.84	1.96
25% Percentile	13.6	4.53	3.05	1.98	13.1	3.84	3.08	2.41
Median	22.2	7.79	4.04	3.46	20.9	6.34	4.38	3.76
75% Percentile	32.6	24	5.77	5.04	29.2	18.3	6.02	6.09
Maximum	68.5	65.2	61.2	14.1	84.6	62.6	22.7	27.4
Mean	24.3	15	5.28	4.21	23.7	12.2	5.04	4.95
Std. Deviation	14.6	15.6	5.56	2.69	16.1	13.5	3.04	3.88

Progesterone	ADT				ADT + Doc			
	BL<2w	BL>2w	wk24	EOS	BL<2w	BL>2w	wk24	EOS
Minimum	0.014	0.013	0.01	0.009	0.01	0.01	0.01	0.01
25% Percentile	0.033	0.02	0.015	0.015	0.02	0.01	0.016	0.018
Median	0.046	0.0275	0.02	0.02	0.03	0.02	0.02	0.021
75% Percentile	0.062	0.044	0.0303	0.029	0.05	0.0325	0.026	0.032
Maximum	0.591	0.101	0.25	6.1	0.46	0.17	10.7	16.1
Mean	0.0612	0.0329	0.0274	0.0836	0.0511	0.028	0.0698	0.239
Std. Deviation	0.0794	0.0185	0.0283	0.522	0.07	0.0267	0.683	1.6

Androsterone	ADT				ADT + Doc			
	BL<2w	BL>2w	wk24	EOS	BL<2w	BL>2w	wk24	EOS
Minimum	0.01	0.016	0.01	0.01	0.01	0.01	0.01	0.004
25% Percentile	0.063	0.0493	0.033	0.018	0.05	0.03	0.028	0.018
Median	0.117	0.071	0.05	0.038	0.09	0.04	0.0425	0.03
75% Percentile	0.156	0.137	0.0783	0.069	0.12	0.08	0.066	0.059
Maximum	0.411	0.322	0.198	0.164	0.33	0.26	0.209	0.179
Mean	0.13	0.103	0.0583	0.0474	0.098	0.0646	0.0515	0.0427
Std. Deviation	0.0845	0.0783	0.0366	0.0345	0.0636	0.0586	0.0341	0.0351

Estrone (E1)	ADT				ADT + Doc			
	BL<2w	BL>2w	wk24	EOS	BL<2w	BL>2w	wk24	EOS
Minimum	4.44	6.56	1.96	1.96	3.1	1.96	1.96	1.38
25% Percentile	18.8	16.8	11.7	8.57	16.9	9.9	13.4	9.32
Median	29	23.8	17.00	14.3	24.8	17	20.5	17.4
75% Percentile	44.9	44.2	24.1	22.2	34.5	26.3	29	26.7
Maximum	91.1	108	81.9	75.7	83.3	96.1	79.8	244
Mean	33.6	32.2	19.7	17.1	27.4	21.5	22.7	21.3
Std. Deviation	20.1	20.9	12.6	12.7	15.4	18	12.8	26.4

Pregnenolone	ADT				ADT + Doc			
	BL<2w	BL>2w	wk24	EOS	BL<2w	BL>2w	wk24	EOS
Minimum	0.149	0.197	0.033	0.018	0.07	0.05	0.068	0.01
25% Percentile	0.468	0.434	0.31	0.211	0.32	0.2	0.322	0.22
Median	0.71	0.628	0.53	0.387	0.45	0.275	0.467	0.42
75% Percentile	1.18	0.978	0.88	0.804	0.785	0.64	0.736	0.745
Maximum	3.45	2.01	8.11	8.33	4.8	4.57	46	23.5
Mean	0.896	0.767	0.698	0.693	0.708	0.532	0.815	1.02
Std. Deviation	0.599	0.45	0.712	1.07	0.824	0.69	2.95	2.89

Dehydroepiandrosterone (DHEA)	ADT				ADT + Doc			
	BL<2w	BL>2w	wk24	EOS	BL<2w	BL>2w	wk24	EOS
Minimum	0.043	0.132	0.012	0.01	0.1	0.06	0.045	0.01
25% Percentile	0.738	0.913	0.621	0.334	0.45	0.438	0.509	0.265
Median	1.09	1.47	1.26	0.796	0.78	0.63	0.984	0.612
75% Percentile	1.78	2.13	1.92	1.66	1.24	0.973	1.46	1.33
Maximum	6.45	5.05	9.16	5.73	6.97	6.53	6.97	4.37
Mean	1.48	1.64	1.51	1.19	1.05	0.936	1.21	0.889
Std. Deviation	1.28	1.13	1.29	1.14	1.16	1.11	1.04	0.826

Androstenedione (AED)	ADT				ADT + Doc			
	BL<2w	BL>2w	wk24	EOS	BL<2w	BL>2w	wk24	EOS
Minimum	0.016	0.084	0.01	0.01	0.05	0.02	0.01	0.01
25% Percentile	0.33	0.265	0.214	0.147	0.195	0.09	0.186	0.136
Median	0.445	0.449	0.32	0.283	0.29	0.16	0.272	0.24
75% Percentile	0.629	0.646	0.439	0.425	0.465	0.323	0.407	0.401
Maximum	1.47	1.39	1.29	2	2	1.09	2.73	1.04
Mean	0.531	0.488	0.352	0.314	0.382	0.244	0.334	0.298
Std. Deviation	0.312	0.286	0.211	0.25	0.303	0.223	0.251	0.24

Supplementary Data 4 – The steroid synthetic pathway

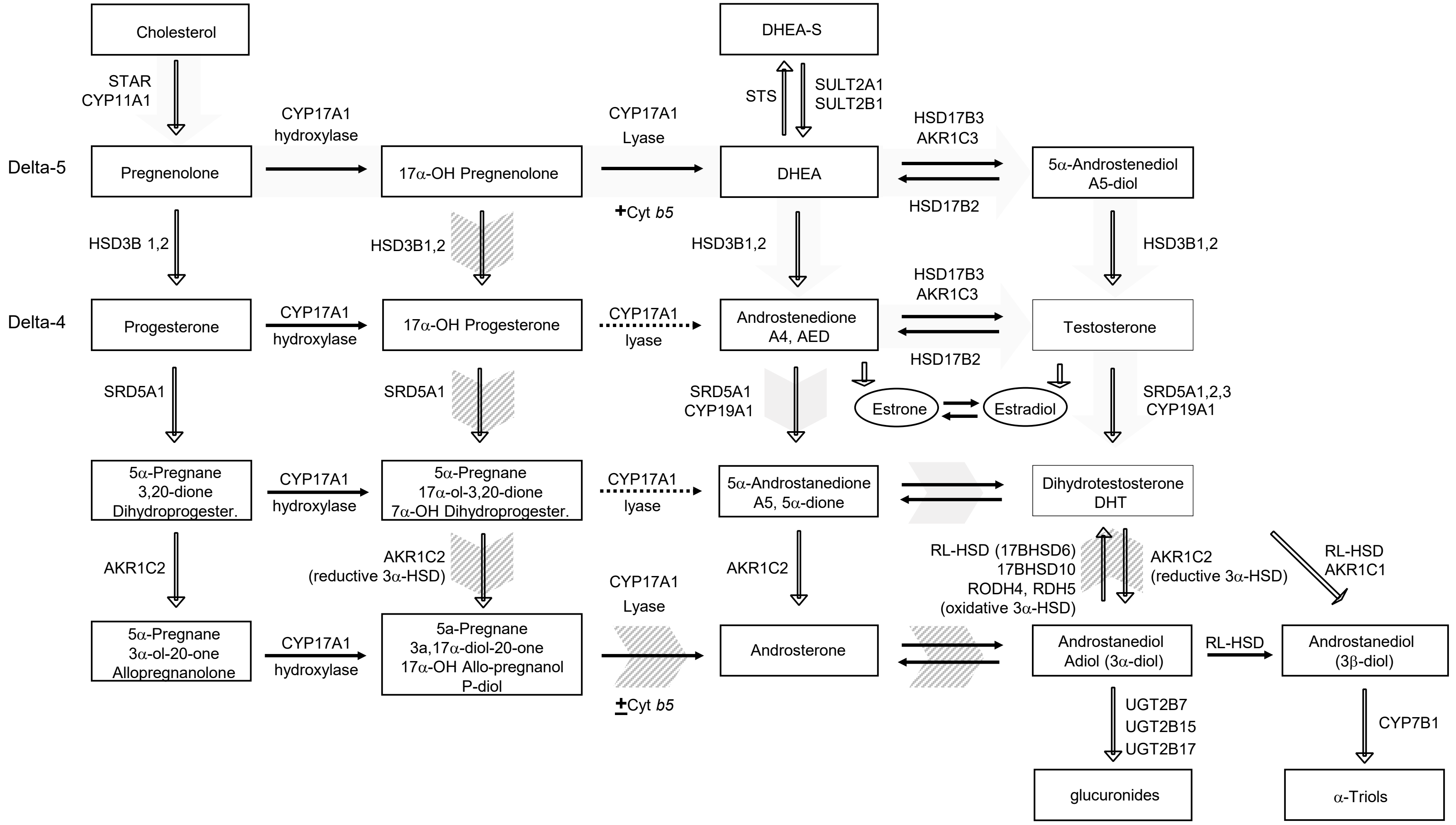

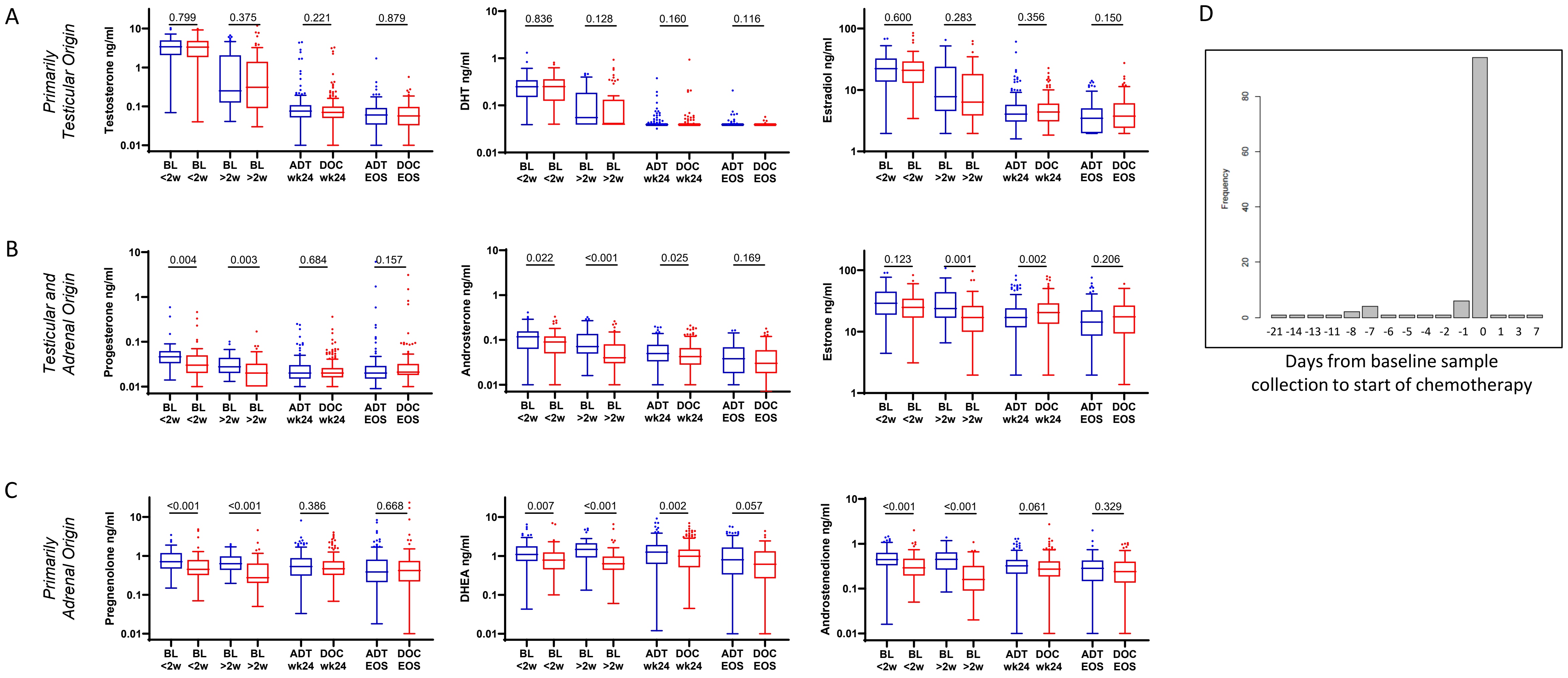

Supplementary Data 5 – Intragroup comparison of steroid levels at each timepoint in ADT alone (Blue) vs ADT + Docetaxel (Red) treatment arms. Steroids are grouped according to whether they are primarily A) of testicular origin (testosterone, DHT, and estradiol), B) of mixed testicular and adrenal origin (progesterone, androsterone, and estrone), or C) of primarily adrenal origin (pregnenolone, DHEA, and AED). Data are shown as box and-whisker plots, where horizontal lines indicate median values; white boxes denote the 75th (upper margin) and 25th percentiles (lower margin), and upper and lower bars indicate the minimum and maximum values, respectively. D) Frequency distribution of patients based on number of days from baseline sample collection to start *of chemotherapy. BL – baseline; wk – week; ADT – androgen deprivation therapy; Doc – docetaxel; EOS – end of study.*

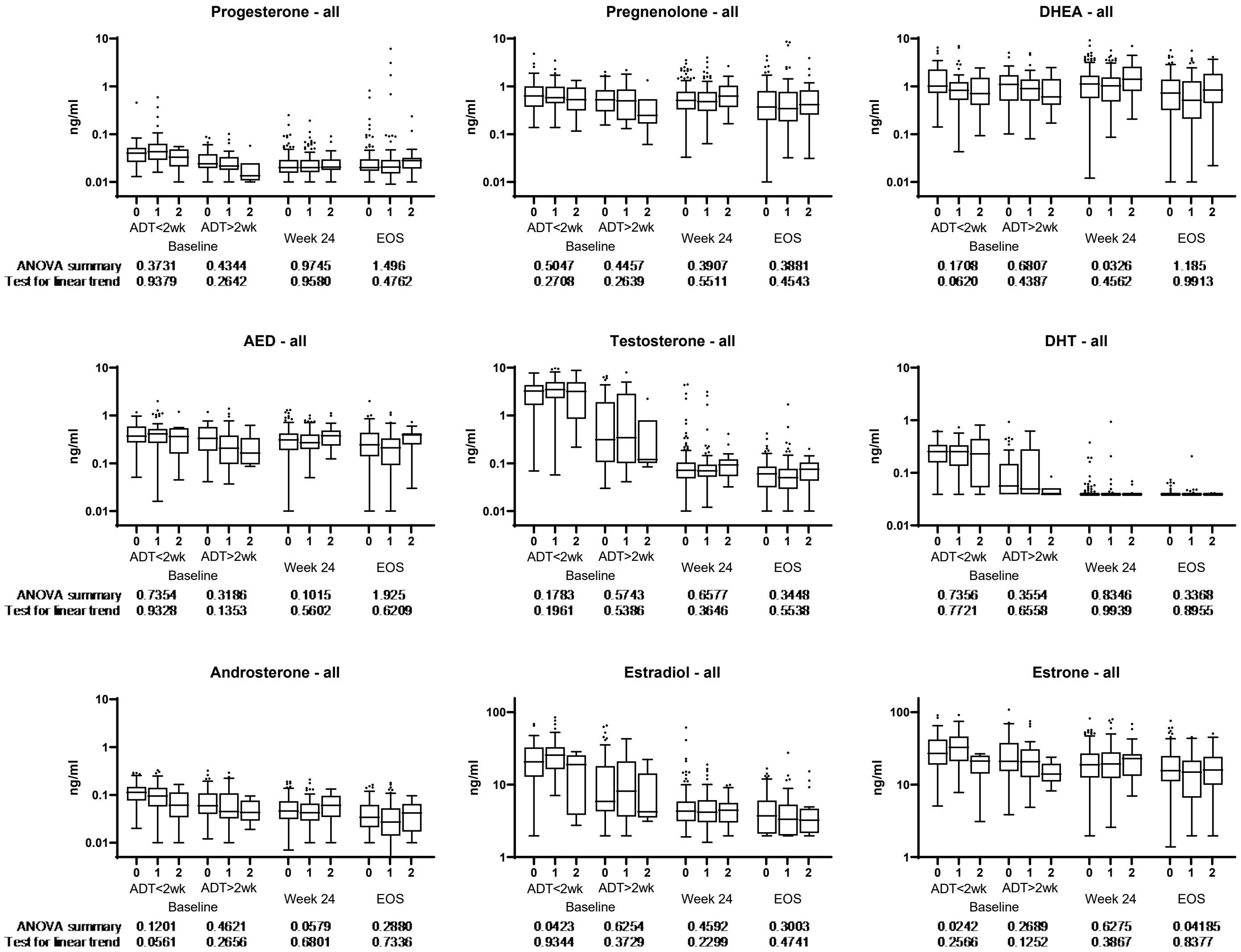

Supplementary Data 6 – Distribution of Steroid Levels by HSD3B1 status. Levels of the indicated metabolite are shown at baseline, at week 24 or at end of study (EOS), for patients with 0, 1, or 2 copies of the variant HSD3B1 allele. Baseline data are divided into whether patients had started ADT less than or more than two weeks prior to sample collection. Data are shown as box and-whisker plots, where horizontal lines indicate median values; white boxes denote the 75th (upper margin) and 25th percentiles (lower margin), and upper and lower bars indicate the minimum and maximum values, respectively. ADT – androgen deprivation therapy; EOS – end of study.

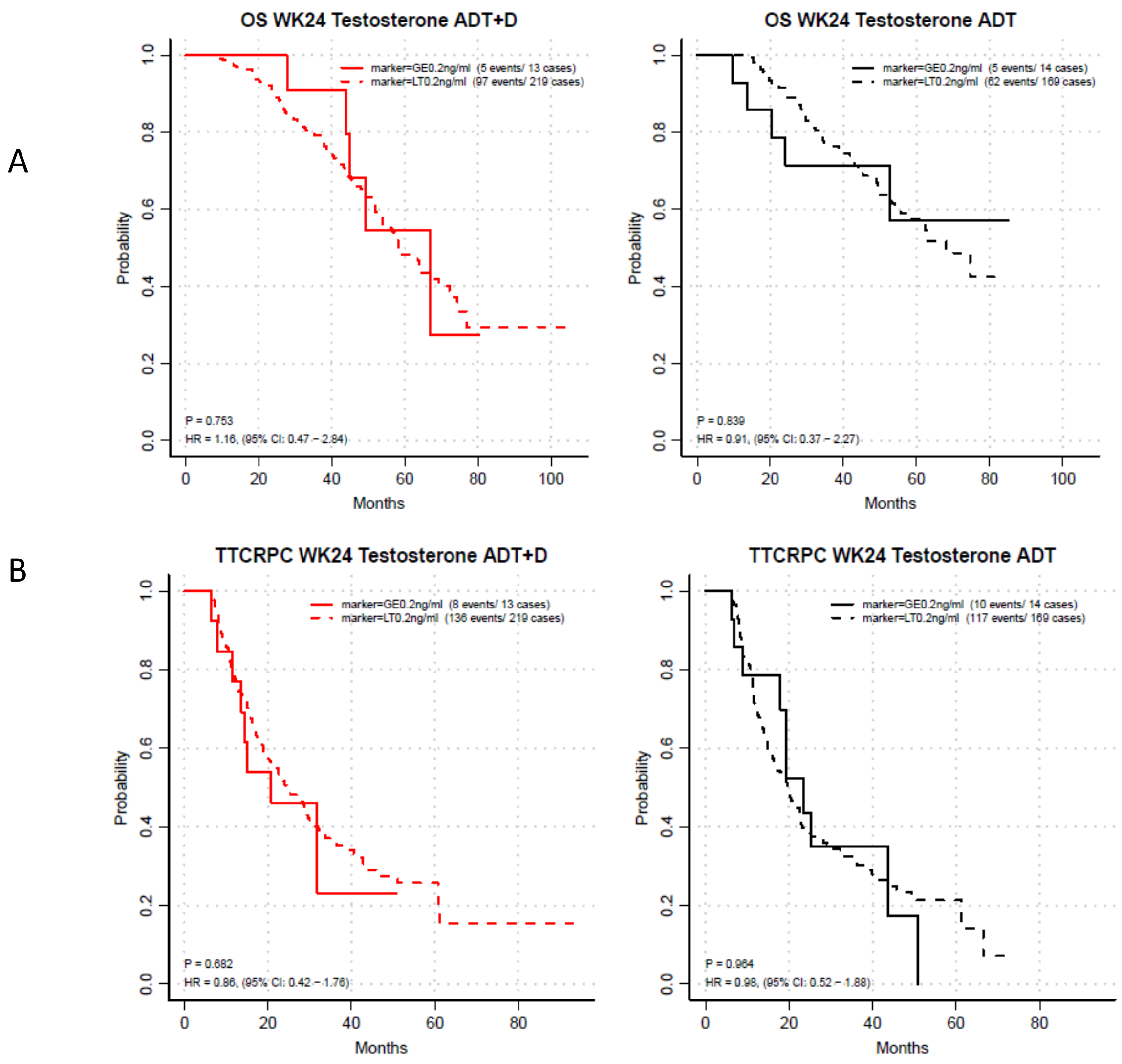

Supplementary Data 7 –Survival curves associated with having testosterone levels above or below 20ng/dl at week 24 of treatment **.** A) OS at week 24 for patients in the ADT + D arm (left) and ADT alone treatment arms (right). B) TTCRPC at week 24 for patients in the ADT + D arm (left) and ADT alone treatment arms (right). OS – overall survival, TTCRPC – time to castration resistant prostate cancer; ADT – androgen deprivation therapy; D – docetaxel, wk - week.

Supplementary Data 8 – Marker distribution for serum steroid levels

Marker distribution at week 24 (arms combined). The Q1 cutoff is ≤ Q1 vs > Q1

(ng/ml)	Min.	1st Qu.	Median	Mean	3rd Qu.	Max.	NA's
Androstenedione	0.0050	0.1980	0.2925	0.3425	0.4250	2.7330	120
Androsterone	0.0070	0.0300	0.0460	0.0547	0.0710	0.2090	120
DHEA	0.0120	0.5613	1.0905	1.3527	1.6575	9.1630	120
DHT	0.0320	0.0390	0.0390	0.0446	0.0390	0.9350	120
Pregnenolone	0.0330	0.3185	0.4955	0.7593	0.7978	46.0330	120
Progesterone	0.0100	0.0160	0.0200	0.0497	0.0283	10.7300	120
Testosterone	0.0100	0.0510	0.0725	0.1475	0.1050	4.4640	120
E2	1.6000	3.0600	4.1900	5.1551	5.8500	61.2000	146
E1	1.9600	12.3050	18.8200	21.3229	26.6750	81.8500	146

Marker distribution at CRPC (arms combined). The Q1 cutoff is ≤ Q1 vs > Q1.

(ng/ml)	Min.	1st Qu.	Median	Mean	3rd Qu.	Max.	NA's
Androstenedione	0.002	0.143	0.26	0.307	0.413	1.997	336
Androsterone	0.004	0.018	0.036	0.045	0.063	0.179	336
DHEA	0.001	0.305	0.732	1.063	1.578	5.726	336
DHT	0.039	0.039	0.039	0.04	0.039	0.207	336
Pregnenolone	0.003	0.217	0.405	0.829	0.763	23.479	336
Progesterone	0.009	0.017	0.021	0.148	0.03	16.133	336
Testosterone	0.01	0.034	0.06	0.08	0.092	1.7	336
E2	1.96	2.145	3.66	4.517	5.355	27.44	354
E1	1.38	9.025	15.63	18.868	24.04	244.28	354

Supplementary Data 9 – Unadjusted HR for overall survival (OS), time to CRPC (TTCRPC) and survival from CRPC for treatment with ADT+Doc vs ADT alone by quartile of steroid level and disease group

Overall Survival (OS)	All Patients								High Volume								Low Volume							
	Q1				Q234				Q1				Q234				Q1				Q234			
serum at week 24	HR	95% CI		P-val	HR	95% CI		P-val	HR	95% CI		P-val	HR	95% CI		P-val	HR	95% CI		P-val	HR	95% CI		P-val
Pregnenolone	0.62	0.36	1.06	0.078	1.05	0.77	1.44	0.761	0.53	0.28	1.00	0.046	0.82	0.57	1.19	0.298	0.72	0.25	2.09	0.548	1.50	0.81	2.77	0.192
Progesterone	0.68	0.41	1.13	0.131	1.03	0.75	1.43	0.855	0.69	0.38	1.25	0.221	0.74	0.50	1.08	0.113	0.59	0.22	1.59	0.29	1.70	0.91	3.19	0.095
DHEA	0.61	0.35	1.05	0.07	1.04	0.76	1.42	0.801	0.69	0.37	1.32	0.26	0.73	0.50	1.05	0.089	0.45	0.15	1.30	0.13	1.75	0.95	3.22	0.071
Androstenedione	0.44	0.26	0.76	0.002	1.15	0.84	1.58	0.375	0.47	0.25	0.89	0.018	0.83	0.58	1.21	0.334	0.48	0.17	1.36	0.158	1.69	0.91	3.12	0.091
Testosterone	0.54	0.31	0.92	0.021	1.09	0.80	1.50	0.579	0.58	0.30	1.11	0.095	0.78	0.54	1.12	0.174	0.39	0.15	1.06	0.055	2.04	1.06	3.95	0.031
DHT	0.88	0.66	1.17	0.379	1.25	0.48	3.23	0.651	0.73	0.53	1.02	0.066	0.75	0.24	2.36	0.627	1.15	0.67	1.99	0.616	2.11	0.19	23.90	0.539
Androsterone	0.66	0.39	1.11	0.115	1.03	0.75	1.41	0.88	0.64	0.35	1.18	0.149	0.77	0.53	1.11	0.16	0.77	0.29	2.07	0.599	1.47	0.79	2.73	0.225
E2	0.95	0.55	1.64	0.85	0.93	0.67	1.28	0.645	0.88	0.46	1.70	0.71	0.64	0.44	0.93	0.018	0.91	0.33	2.51	0.85	1.62	0.86	3.07	0.135
E1	0.68	0.38	1.21	0.186	1.03	0.75	1.43	0.848	0.76	0.38	1.52	0.438	0.66	0.45	0.96	0.029	0.51	0.17	1.50	0.211	2.03	1.05	3.92	0.031

HR<1 favors ADT+Doc; HR>1 favors ADT alone

TTCRPC	All Patients								High Volume								Low Volume							
	Q1				Q234				Q1				Q234				Q1				Q234			
serum at week 24	HR	95% CI		P-val	HR	95% CI		P-val	HR	95% CI		P-val	HR	95% CI		P-val	HR	95% CI		P-val	HR	95% CI		P-val
Pregnenolone	0.70	0.43	1.16	0.163	0.84	0.64	1.11	0.223	0.62	0.34	1.13	0.113	0.74	0.53	1.04	0.084	0.69	0.28	1.71	0.414	0.81	0.50	1.31	0.385
Progesterone	0.74	0.47	1.16	0.185	0.83	0.62	1.10	0.188	0.75	0.43	1.30	0.299	0.71	0.50	1.01	0.052	0.58	0.25	1.35	0.2	0.81	0.50	1.32	0.4
DHEA	0.77	0.46	1.27	0.303	0.85	0.65	1.11	0.233	0.82	0.45	1.50	0.525	0.67	0.48	0.94	0.019	0.46	0.16	1.27	0.122	0.94	0.59	1.49	0.785
Androstenedione	0.58	0.35	0.95	0.03	0.96	0.73	1.26	0.743	0.63	0.35	1.14	0.124	0.76	0.54	1.06	0.108	0.40	0.15	1.08	0.061	0.99	0.62	1.58	0.956
Testosterone	0.61	0.38	0.97	0.036	0.91	0.69	1.20	0.507	0.63	0.34	1.14	0.12	0.75	0.53	1.05	0.093	0.39	0.17	0.93	0.028	1.03	0.63	1.68	0.912
DHT	0.81	0.63	1.05	0.107	0.87	0.41	1.87	0.724	0.72	0.53	0.99	0.039	0.63	0.25	1.57	0.318	0.78	0.50	1.22	0.272	0.98	0.24	3.92	0.974
Androsterone	0.77	0.46	1.28	0.308	0.85	0.65	1.12	0.243	0.75	0.40	1.38	0.349	0.71	0.51	0.99	0.043	0.73	0.29	1.82	0.495	0.83	0.51	1.35	0.455
E2	0.74	0.45	1.22	0.238	0.90	0.68	1.19	0.462	0.79	0.43	1.43	0.425	0.73	0.51	1.04	0.083	0.56	0.22	1.43	0.221	0.99	0.61	1.60	0.952
E1	0.56	0.34	0.95	0.028	0.98	0.74	1.30	0.889	0.68	0.37	1.27	0.228	0.73	0.51	1.05	0.09	0.35	0.13	0.95	0.032	1.13	0.70	1.84	0.612

HR<1 favors ADT+Doc; HR>1 favors ADT alone

Survival from CRPC	All Patients								High Volume								Low Volume							
	Q1				Q234				Q1				Q234				Q1				Q234			
serum at progression	HR	95% CI		P-val	HR	95% CI		P-val	HR	95% CI		P-val	HR	95% CI		P-val	HR	95% CI		P-val	HR	95% CI		P-val
Pregnenolone	1.10	0.65	1.89	0.720	1.10	0.77	1.57	0.593	1.01	0.56	1.82	0.968	0.89	0.59	1.33	0.557	1.35	0.33	5.49	0.676	1.59	0.76	3.36	0.218
Progesterone	0.96	0.52	1.77	0.896	1.12	0.80	1.58	0.502	0.72	0.35	1.51	0.388	0.96	0.66	1.41	0.848	3.48	0.86	14.10	0.063	1.30	0.60	2.83	0.51
DHEA	0.77	0.44	1.35	0.352	1.20	0.85	1.70	0.296	0.68	0.36	1.28	0.232	0.97	0.65	1.43	0.865	0.89	0.23	3.45	0.865	2.02	0.94	4.34	0.066
Androstenedione	1.03	0.60	1.77	0.911	1.08	0.76	1.54	0.66	0.97	0.53	1.80	0.93	0.87	0.59	1.30	0.503	1.38	0.39	4.82	0.615	1.69	0.77	3.69	0.184
Testosterone	1.03	0.59	1.79	0.931	1.10	0.77	1.56	0.605	0.92	0.50	1.69	0.795	0.87	0.58	1.30	0.49	1.14	0.22	6.00	0.876	1.83	0.88	3.81	0.1
DHT	1.09	0.80	1.47	0.592	0.39	0.05	3.11	0.356	0.93	0.66	1.31	0.671	0.00	0.00	Inf	0.06	1.52	0.79	2.95	0.208				
Androsterone	0.84	0.49	1.45	0.529	1.20	0.84	1.71	0.314	0.87	0.47	1.62	0.655	0.94	0.63	1.40	0.757	0.82	0.26	2.55	0.727	2.14	0.94	4.87	0.064
E2	1.15	0.64	2.06	0.653	1.15	0.80	1.66	0.436	0.72	0.36	1.46	0.356	1.00	0.67	1.50	0.987	4.28	1.00	18.42	0.035	1.40	0.62	3.21	0.418
E1	1.07	0.61	1.89	0.81	1.12	0.78	1.62	0.537	0.66	0.34	1.28	0.22	0.99	0.66	1.49	0.961	3.55	0.82	15.32	0.072	1.43	0.63	3.23	0.386

HR<1 favors ADT+Doc; HR>1 favors ADT alone

Supplementary Data 10. Unadjusted HR for overall survival (OS), time to CRPC (TTCRPC) and survival from CRPC for lowest (Q1) vs 3 highest (Q234) quartiles of steroid levels by treatment arm and disease volume

Overall Survival (OS)	All Patients								High Volume								Low Volume							
	ADT				ADT+Doc				ADT				ADT+Doc				ADT				ADT+Doc			
serum at week 24	HR	95% CI		P-val	HR	95% CI		P-val	HR	95% CI		P-val	HR	95% CI		P-val	HR	95% CI		P-val	HR	95% CI		P-val
Pregnenolone	1.36	0.88	2.08	0.164	0.79	0.50	1.26	0.324	1.36	0.82	2.25	0.229	0.87	0.51	1.48	0.605	1.41	0.61	3.25	0.415	0.66	0.27	1.62	0.359
Progesterone	1.28	0.84	1.96	0.253	0.85	0.56	1.29	0.456	0.98	0.59	1.63	0.943	0.98	0.61	1.58	0.942	1.94	0.86	4.35	0.103	0.56	0.24	1.32	0.179
DHEA	1.42	0.90	2.23	0.132	0.82	0.53	1.27	0.372	1.04	0.60	1.80	0.889	0.95	0.58	1.56	0.834	2.44	1.06	5.62	0.03	0.57	0.23	1.40	0.212
Androstenedione	1.65	1.06	2.58	0.025	0.66	0.43	1.03	0.063	1.23	0.73	2.07	0.427	0.69	0.41	1.16	0.162	2.55	1.07	6.10	0.028	0.65	0.29	1.48	0.3
Testosterone	1.49	0.97	2.29	0.069	0.74	0.47	1.17	0.198	1.08	0.63	1.86	0.772	0.79	0.47	1.33	0.369	2.80	1.29	6.06	0.006	0.65	0.26	1.61	0.35
DHT	1.61	0.81	3.19	0.169	1.16	0.56	2.38	0.689	1.25	0.54	2.90	0.599	1.25	0.58	2.72	0.568	1.61	0.48	5.38	0.433	0.97	0.13	7.25	0.976
Androsterone	1.38	0.88	2.14	0.158	0.90	0.59	1.38	0.637	1.18	0.70	1.98	0.528	0.98	0.60	1.61	0.938	1.68	0.70	4.00	0.238	0.85	0.38	1.88	0.682
E2	1.05	0.66	1.65	0.843	1.09	0.70	1.70	0.691	0.86	0.50	1.48	0.591	1.24	0.74	2.09	0.413	1.47	0.63	3.44	0.375	0.85	0.36	1.98	0.702
E1	1.15	0.75	1.78	0.518	0.74	0.45	1.23	0.241	0.80	0.48	1.34	0.4	0.90	0.50	1.64	0.742	1.92	0.85	4.33	0.109	0.51	0.19	1.34	0.161

HR<1 favors q1 (lowest quartile of steroid level), HR>1 favors q234 (highest 3 quartiles)

TTCRPC	All Patients								High Volume								Low Volume							
	ADT				ADT+Doc				ADT				ADT+Doc				ADT				ADT+Doc			
serum at week 24	HR	95% CI		P-val	HR	95% CI		P-val	HR	95% CI		P-val	HR	95% CI		P-val	HR	95% CI		P-val	HR	95% CI		P-val
Pregnenolone	0.97	0.65	1.44	0.861	0.80	0.54	1.19	0.269	0.89	0.54	1.46	0.634	0.79	0.50	1.26	0.317	1.00	0.52	1.92	0.994	0.78	0.36	1.69	0.527
Progesterone	1.00	0.68	1.48	0.987	0.91	0.63	1.31	0.605	0.89	0.55	1.45	0.633	0.95	0.62	1.44	0.797	1.08	0.56	2.09	0.825	0.75	0.36	1.55	0.437
DHEA	0.96	0.63	1.47	0.856	0.81	0.55	1.18	0.272	0.68	0.40	1.16	0.157	0.90	0.59	1.39	0.642	1.19	0.57	2.47	0.638	0.51	0.23	1.17	0.106
Androstenedione	1.08	0.71	1.65	0.714	0.60	0.41	0.88	0.009	0.73	0.44	1.22	0.225	0.65	0.41	1.02	0.056	1.38	0.64	2.97	0.409	0.49	0.23	1.04	0.059
Testosterone	1.26	0.85	1.86	0.249	0.79	0.54	1.16	0.223	0.88	0.53	1.49	0.64	0.82	0.53	1.27	0.372	1.81	0.99	3.32	0.052	0.65	0.30	1.42	0.278
DHT	0.91	0.55	1.48	0.693	0.88	0.48	1.63	0.692	0.95	0.50	1.80	0.87	1.08	0.52	2.23	0.833	0.86	0.40	1.87	0.708	0.58	0.18	1.90	0.361
Androsterone	0.88	0.57	1.36	0.569	0.77	0.53	1.12	0.167	0.69	0.40	1.19	0.179	0.79	0.50	1.23	0.287	1.00	0.48	2.07	0.997	0.77	0.38	1.54	0.453
E2	1.13	0.75	1.70	0.551	0.95	0.64	1.41	0.792	1.12	0.67	1.87	0.654	1.29	0.81	2.04	0.28	1.05	0.53	2.07	0.892	0.56	0.26	1.23	0.142
E1	1.21	0.82	1.79	0.342	0.65	0.42	1.01	0.052	0.96	0.59	1.58	0.883	0.89	0.53	1.48	0.646	1.31	0.69	2.51	0.406	0.38	0.16	0.91	0.025

HR<1 favors q1 (lowest quartile of steroid level), HR>1 favors q234 (highest 3 quartiles)

Survival from CRPC	All Patients								High Volume								Low Volume							
	ADT				ADT+Doc				ADT				ADT+Doc				ADT				ADT+Doc			
serum at progression	HR	95% CI		P-val	HR	95% CI		P-val	HR	95% CI		P-val	HR	95% CI		P-val	HR	95% CI		P-val	HR	95% CI		P-val
Pregnenolone	2.16	1.43	3.25	0.000	2.22	1.35	3.65	0.001	2.08	1.32	3.29	0.001	2.19	1.25	3.82	0.005	1.97	0.66	5.88	0.216	1.99	0.61	6.49	0.248
Progesterone	1.05	0.68	1.61	0.824	0.92	0.53	1.59	0.757	1.12	0.70	1.78	0.644	0.85	0.43	1.67	0.63	0.54	0.16	1.84	0.318	1.26	0.45	3.53	0.659
DHEA	1.57	1.02	2.41	0.038	0.99	0.60	1.64	0.977	1.56	0.95	2.55	0.076	0.98	0.56	1.72	0.956	2.11	0.85	5.24	0.099	0.79	0.22	2.86	0.723
Androstenedione	1.56	1.03	2.38	0.034	1.53	0.93	2.52	0.094	1.61	1.01	2.58	0.046	1.58	0.90	2.77	0.111	1.66	0.64	4.29	0.292	1.42	0.48	4.26	0.525
Testosterone	1.52	1.00	2.30	0.049	1.46	0.88	2.44	0.142	1.53	0.95	2.45	0.077	1.50	0.86	2.62	0.15	1.70	0.66	4.41	0.266	1.18	0.26	5.29	0.832
DHT	1.28	0.64	2.53	0.485	2.71	0.38	19.61	0.302	1.02	0.49	2.13	0.955	2.90	0.40	20.83	0.27	2.58	0.35	19.23	0.337				
Androsterone	1.71	1.13	2.58	0.01	1.17	0.71	1.92	0.538	1.55	0.98	2.46	0.061	1.30	0.73	2.29	0.372	2.05	0.79	5.29	0.13	0.71	0.24	2.11	0.533
E2	1.34	0.88	2.05	0.172	1.30	0.75	2.25	0.345	1.47	0.92	2.34	0.103	1.07	0.56	2.03	0.844	1.07	0.35	3.31	0.909	3.36	1.08	10.42	0.027
E1	1.27	0.81	1.97	0.295	1.25	0.74	2.08	0.402	1.76	1.08	2.86	0.021	1.23	0.69	2.21	0.485	0.61	0.17	2.16	0.438	1.31	0.44	3.89	0.622

HR<1 favors q1 (lowest quartile of steroid level), HR>1 favors q234 (highest 3 quartiles)
